# Anterior Cervical Spondylosis Surgical Interventions are Associated with Improved Lordosis and Neurological Outcomes at Latest Follow up: A Meta-analysis

**DOI:** 10.1038/s41598-017-04311-6

**Published:** 2017-06-30

**Authors:** Zengdong Meng, Jing Yu, Chong Luo, Xia Liu, Wei Jiang, Lehua Yu, Rongzhong Huang

**Affiliations:** 1 Department of Orthopaedics, First People’s Hospital of YunNan Province, YunNan, P. R. China; 2 Department of Rehabilitation Medicine, The Second Affiliated Hospital of Chongqing Medical University, Chongqing, P. R. China; 3 Department of Preventive Medicine, Keck School of Medicine, University of Southern California, Los Angeles, CA P. R. China; 4 Shanghai Key Laboratory of Forensic Medicine, Institute of Forensic Science, Ministry of Justice, Shanghai, P.R. China

## Abstract

Aim of this study was to evaluate the effect of cervical spondylosis surgery on cervical lordosis and to identify factors affecting the change by latest follow-up. Literature search was carried out in electronic databases and study selection followed *a priori* eligibility criteria. Random effects meta-analyses were performed to estimate effect size/s of change in lordosis after surgery (at latest follow-up) and metaregression analyses were performed to identify factors affecting this change. Nineteen studies (1845 patients; age 55.18 [95% CI: 54.78, 55.57] years; 60.99 [60.63, 61.36] % males; follow-up 25.59 [25.20, 25.99] months) were included. Whereas, corpectomy (4.06 [2.65, 5.46] degree; p < 0.00001) and discectomy (4.59 [2.07, 7.11] degree; p < 0.00001) were associated with increase, laminectomy (−1.87 [−8.40, 4.66] degree; p = 0.57) and laminoplasty (0.25 [−1.07, 1.56] degree; p = 0.711) were not associated with significant change in lordosis at latest follow-up. Change in Japanese Orthopedic Association (JOA)/modified JOA (mJOA) score at latest follow-up was also significantly (p = 0.0005) higher in anterior than in posterior surgery group. Change in lordosis at latest follow-up had significant positive relationship with follow-up duration but had significant inverse associations with age, male gender, and preoperative JOA/mJOA score, independently. In posterior surgery subjects, after adjusting for age and gender, preoperative JOA/mJOA score was significantly inversely related to change in lordosis.

## Introduction

Cervical spondylosis is the osteoarthritic degeneration of cervical spine components such as uncovertebral and facet joints and intervertebral discs causing several motor and sensory dysfunctions. Pathophysiology is multi-factorial that may include the formation of osteophyte and/or chondro-osseous spurs, disc herniation, and hypertrophy of facet joints and/or ligaments which affect canal diameter and sagittal mobility^1–3^.

Cervical spondylosis manifests as myelopathy, radiculopathy or radiculomyelopathy which requires radiological diagnosis^4–6^. Symptoms may include weakness, gait instability, decreased dexterity, gut and urinary problems, spasticity, axial pain in the neck and arm regions, headache, blurred vision, tinnitus, palpitations, hypomnesia, and facial flushing and sweating^7, 8^.

Conservative management is usually restricted to cases with mild and moderate symptoms^9, 10^ and more serious cases require surgery involving either anterior, posterior, or circumferential decompression^11, 12^ depending on number of diseased segments, sagittal alignment, stenosis morphology, history of prior surgery, and bone quality^1^. Early surgical intervention has potential for the prevention of significant morbidity and mortality in patients with progressive instability and neurologic deficit^13^.

A large number of studies have reported the outcomes of various surgical interventions in cervical spondylotic myelopathy and/or radiculopathy patients with follow-up of up to 15 years. Although, many of these studies have also reported outcomes with regards to the change in cervical lordosis but variability in the effect size provides impetus for a systematic review to refine the evidence. There is no previous systematic review of studies which examined the change in lordosis after surgical intervention. Therefore, aim of the present study was to carry out a systematic review of relevant studies and to perform a meta-analysis of the of the change in lordosis and neurological outcomes at the latest follow-up reported in individual studies and to identify the factors affecting the change in cervical lordosis.

## Method

This study was performed by following the Cochrane Collaboration’s guidelines^14^ and is reported in accordance with the Preferred Reporting Items for Systematic Reviews and Meta-Analysis (PRISMA) statement^15^.

### Eligibility, meta-analysis endpoint and metaregression analysis variables

Eligible studies of this meta-analysis were those reporting the outcomes of surgical intervention/s in cervical spondylosis patients with a follow-up duration of at least one year and measured change in lordosis preoperatively, after surgery and at latest follow-up.

Meta-analysis endpoints (outcome measures) were: a) the change in lordosis from baseline through the latest follow-up, and b) the change in Japanese Orthopedic Association (JOA)/ modified JOA (mJOA) score from baseline through the latest follow-up. For metaregression analyses, change in lordosis at the latest follow-up (dependent variable) was tested to evaluate the relationships with several explanatory variables including the number of subjects in a study, follow-up duration, age of subjects at the time of surgery, gender (percentage of males), disease duration, percent smokers, percent patients with myelopathy, or radiculopathy, or radiculomyelopathy, percentage of 1, 2, 3, or >3 levels involved, duration of surgery, blood loss during surgery, hospital stay duration, percent incidence of surgical complications, preoperative JOA/mJOA score, preoperative NDI score, preoperative lordosis, preoperative range of motion (ROM), and preoperative neck/arm visual analogue scale (VAS) score.

### Data acquisition

For the acquisition of required data, literature search was undertaken in electronic databases including Embase, Google Scholar, Ovid SP, and PubMed/Medline. For primary search, ‘cervical-spondylosis-surgery’, ‘cervical-spondylotic-myelopathy-surgery’, ‘cervical-spondylotic-radiculopathy-surgery’ and ‘cervical-spondylotic radiculomyelopathy-surgery’ combinations were used. For secondary searches, each of the primary combination was used with several MeSH and keywords including cervical lordosis, cervical curvature, arthroplasty, corpectomy, discectomy, laminectomy, and laminoplasty. Search encompassed original research articles published before October 2015. Additional searches included the bibliographies of retrieved research articles and software suggestions based on retrieved articles.

### Data and analyses

Relevant data including the patients’ demographic/clinical/orthopedic characteristics, surgery type/technique, study population and follow-up characteristics, study design and analyses, outcome assessment and outcomes, and perioperative data were obtained from the retrieved research articles and organized in specialized datasheets.

For the estimation of effect size of the change in lordosis by the latest follow-up, data were either extracted raw from the respective research articles, if provided, or calculated by using baseline and final values given in the study reports. Inverse variance weighted effect sizes of overall (all surgical interventions) and subgroups (surgery type-wise) were obtained under random effects meta-analyses which were carried out in Stata software (version 12; Stata Corporation, College Station, Texas).

Metaregression analyses were also carried out with Stata software by using restricted maximum likelihood (ReML) method. A p value of less than 0.05 was considered to show a significant relationship. Between-study variance was tested with tau^2^ index and the percentage of between-study heterogeneity (outcome inconsistency) was assessed with I^2^ index. Publication bias assessment was carried out with Begg’s funnel plot asymmetry test of meta-analysis of the change of lordosis at the latest follow-up and trim and fill method was used to estimate the number of possible missing studies.

## Results

**Figure 1:**
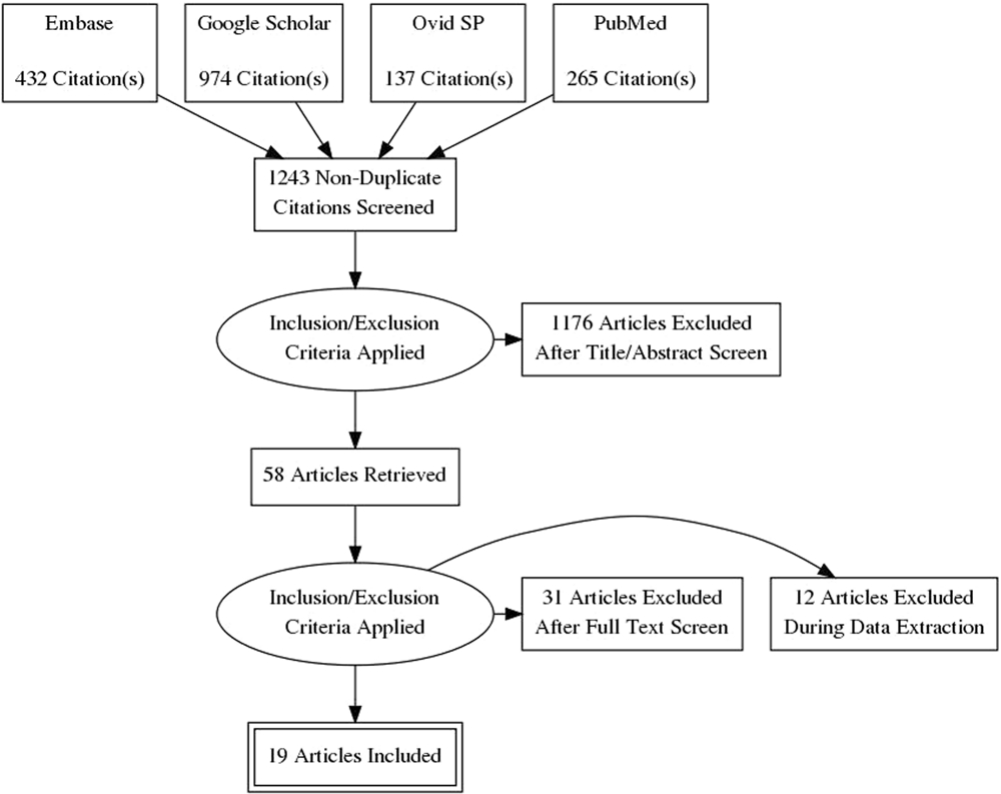
A PRISMA flowchart of study screening and selection process.

**Figure 2:**
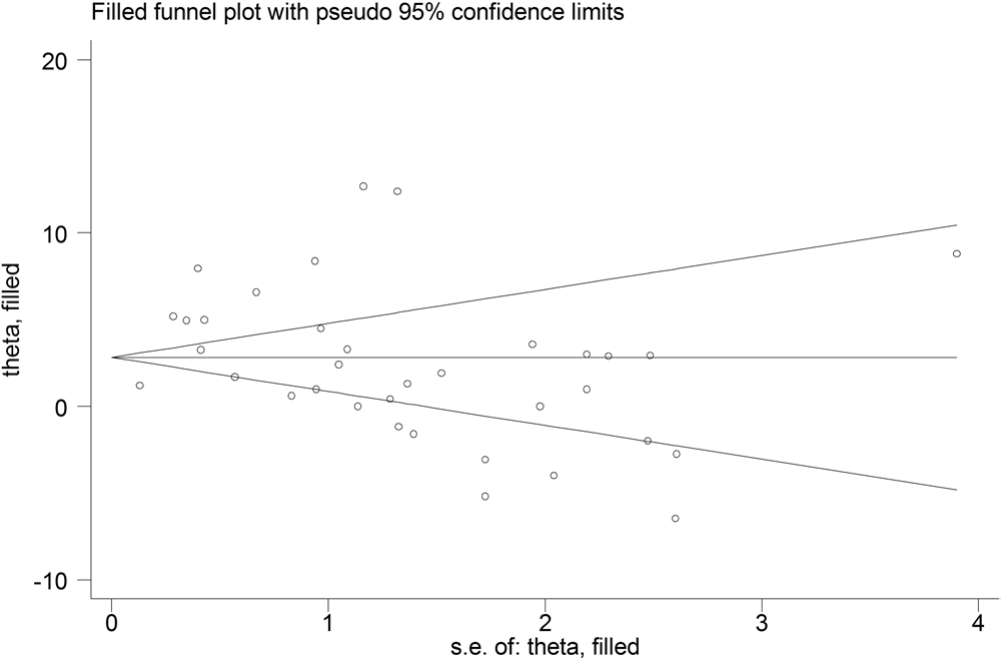
A funnel plot (Y-axis: effect size (theta) and X-axis standard error of the effect size) corresponding to the meta-analysis of the change in lordosis at the latest follow-up showing no significant publication bias.

Nineteen studies (1845 patients) were selected for data acquisition^16–34^ by following the eligibility criteria (Fig. 1). Majority of the included studies had more than one arms and thence the number of datasets attained was 35. There was no significant publication bias (Begg’s Test: adjusted Kendall’s Score (P-Q) = −79 ± 70.42; p = 0.268). Trim and fill method also did not find a significant number of missing studies (Fig. 2).

Important characteristics of the included studies are presented in Table S1 (Supporting information file. Average follow-up duration was 25.59 [95% CI: 25.20, 25.99] (range 12 to 102) months. Average age of the subjects at the time of surgery was 55.18 [54.78, 55.57] years and 60.99 [60.63, 61.36] % of these subjects were male. Average disease duration in these subjects was 27.14 [25.46, 28.83] months. Percentage of subjects with 1, 2, 3, 4 or more than 4 cervical levels involved in surgery were 28 ± 37%, 23 ± 32%, 36 ± 42%, 11 ± 16% and 2 ± 10%, respectively.

Overall, duration of surgery, blood loss during surgery, and hospital stay duration were 133.65 [132.17, 135.14] minutes, 78.86 [75.37, 82.35] milliliter, and 4.41 [4.17, 4.66] days, respectively. There was a significant correlation between the duration of surgery and blood loss (r = 0.657; p < 0.0028), but not between duration of surgery and hospital stay (r = 0.306; p = 0.216), or between blood loss and hospital stay (r = 0.355; p = 0.17).

### Change in lordosis and JOA/mJOA score after cervical spondylosis surgery

**Figure 3:**
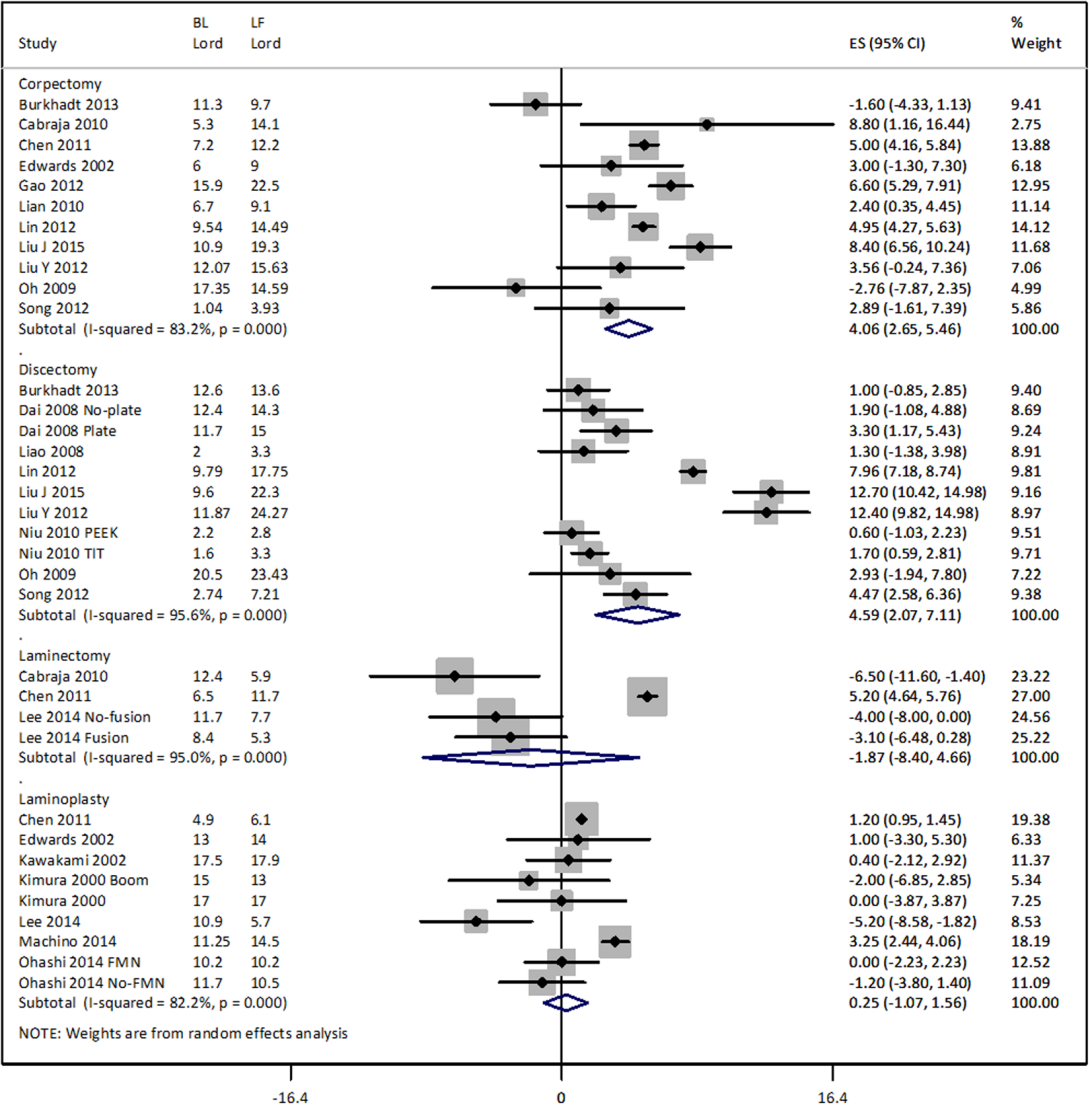
Forest graph showing the surgery type-wise effect sizes of the change in lordosis at the latest follow-up. BL, baseline lordosis (average); LF, Last follow-up lordosis (average); FMN, foraminotomy; PEEK, polyetheretherketone;

Overall, there was a significant difference between anterior and posterior approaches to surgical interventions in changing postoperative lordosis at the latest follow-up (p < 0.00001). Whereas, corpectomy and discectomy were associated with an increase of 4.06 [95% confidence interval: 2.65, 5.46] (p < 0.00001) and 4.59 [2.07, 7.11] degrees (p < 0.00001) respectively, laminectomy (−1.87 [−8.40, 4.66]; p = 0.57) and laminoplasty (0.25 [−1.07, 1.56]; p = 0.711) were not associated with significantly changed lordosis angle at the latest follow-up (Fig. 3).

**Figure 4:**
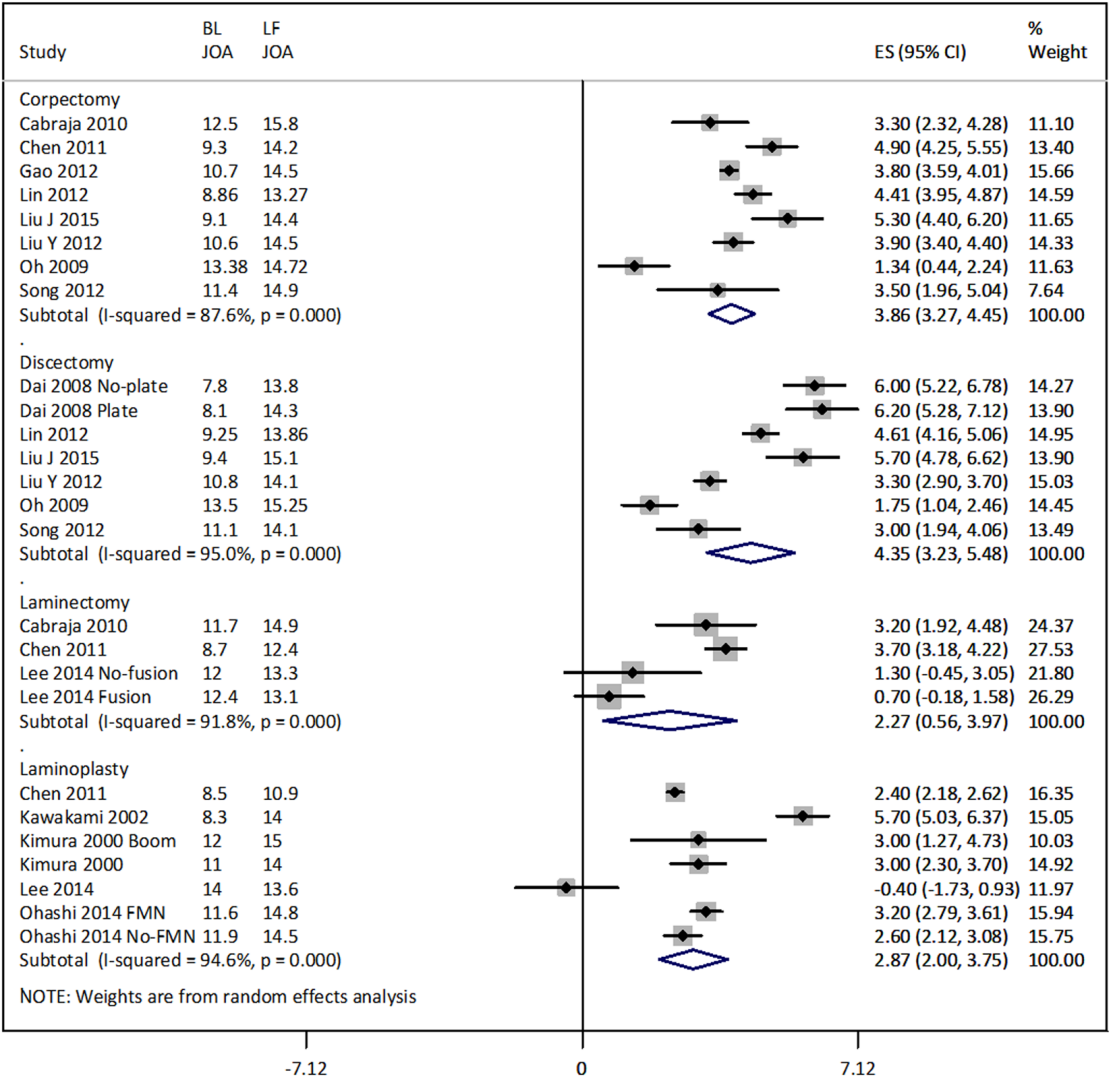
Forest graph showing surgery type-wise effect sizes of the change in JOA/mJOA score at the latest follow-up. BLJOA, baseline JOA/mJOA score (average); LF JOA, last follow-up JOA/mJOA score; FMN, foraminotomy.

Improvement in JOA/mJOA score at the latest follow-up was also significantly (p = 0.0005) higher in anterior surgery (4.08 [3.49, 4.66]; p < 0.00001) than in posterior surgery (2.75 [2.03, 3.48]; p < 0.00001; Fig. 4). Change is JOA/mJOA score at latest follow-up was significantly correlated with the change in lordosis at the latest follow-up (r = 0.575; p = 0.002).

Both the preoperative lordosis and preoperative JOA/mJOA score were not significantly different between anterior and posterior surgery subjects.

**Figure 5:**
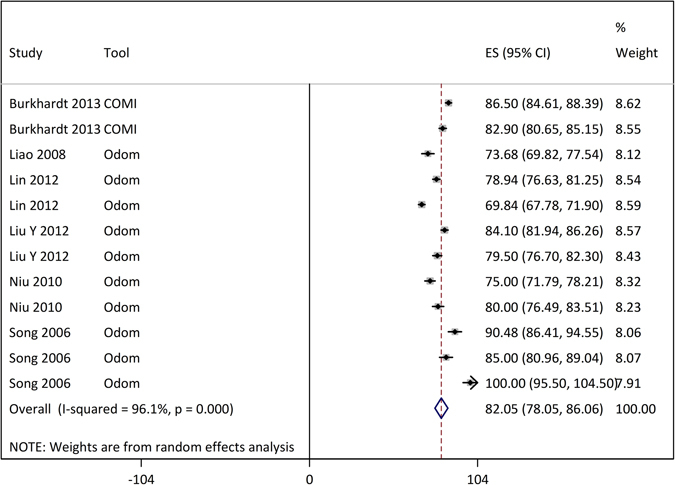
Forest graph showing the overall effect size of patients reported outcomes of the surgery with anterior approaches. ACCF, anterior cervical corpectomy and fusion; ACDF, anterior cervical decompression and fusion; COMI, Core Outcome Measures Index; Odom, Odom grading system; PEEK, polyetheretherketone cage; TIT, titanium cage.

None of the included studies reported SF-36 related patient outcomes; one study used Core Outcome Measures Index (COMI) and four studies used Odom grading for the assessment of patient rating of the surgical outcomes. Overall percentage of patients rating the outcomes as excellent or good was 82.05 [78.05, 86.06] % for anterior surgery (Fig. 5). No data were available with regards to patient-related outcomes for posterior surgery interventions.

### Prognostic factors of change in postoperative lordosis at the latest follow-up

Change in lordosis had a significant positive relationship with the follow-up duration in both anterior (coefficient: 0.058; p = 0.047) and posterior (coefficient: 0.191; p = 0.040) surgical approaches as well in overall surgical interventions (coefficient: 0.085; p = 0.004).

Age was inversely associated with the change in lordosis in overall cervical surgical interventions (coefficient: −0.338; p = 0.005) but not in either anterior surgery (coefficient: −0.204; p = 0.235) or in posterior surgery (coefficient: −0.114; p = 0.595) subgroups. In the overall surgical interventions, percentage of males was inversely associated with the change in lordosis (coefficient: −0.117; p = 0.022) as well as in posterior surgery (coefficient: −0.182; p = 0.057) group. But gender was not significantly associated with the change in lordosis in anterior surgery (coefficient: 0.0009; p = 0.990).

Preoperative JOA/mJOA score was inversely associated with the change in lordosis in the overall surgical interventions (coefficient: −1.301; p = 0.017) as well as in posterior surgery (coefficient: −1.371; p = 0.006) group. However, preoperative JOA/mJOA score was not significantly associated with the change in lordosis in anterior surgery (coefficient: −0.505; p = 0.441).

Change in lordosis at the latest follow-up did not have significant relationship with the preoperative lordosis neither in anterior (coefficient: 0.067; p = 0.696) nor in posterior (coefficient: −0.228; p = 0.389) surgery groups or in overall surgical interventions (coefficient: −0.119; p = 0.445). Neither the percentage of patients with myelopathy, nor radiculopathy or radiculomyelopathy had significant relationships with the change in lordosis at latest follow-up.

In multivariate analyses, none of the independent variables (age, gender or preoperative JOA/mJOA score) was significantly associated with the change in lordosis at the latest follow-up in overall surgical interventions as well as in anterior surgery group. However, in posterior surgery group, preoperative JOA/mJOA score (coefficient: −1.162; p = 0.037) was significantly inversely related to the change in lordosis in multivariate analyses with covariates of age, gender and preoperative JOA/mJOA score.

All outcomes of metaregression analyses are presented in Tables S2–S4.

## Discussion

Anterior surgical approaches are found to be associated with significant increase in lordosis, whereas there was no significant change in lordosis after posterior surgical approaches. Both the surgical approaches were associated with significantly improved JOA/mJOA scores but improvement in JOA/mJOA score was significantly higher in anterior surgery group at latest follow-up. Independently, the change in lordosis had a positive relationship with follow-up duration but had independent inverse relationship with age, percentage of males, and preoperative JOA/mJOA score. However, neither age or gender nor preoperative JOA/mJOA scores were significantly associated with change in lordosis in anterior surgery group. In posterior surgery group, whereas, male gender and preoperative JOA/mJOA score had significantly inverse independent relationships with the change in lordosis but after adjusting for age and gender, only preoperative JOA/mJOA score was inversely associated with the change in lordosis at latest follow-up.

Cervical lordotic curvature can be observed as early as 10 weeks of fetal development arising by the posterior wedging of the cervical disks^35, 36^. In asymptomatic individuals, average cervical lordosis is reported variably; 21.3^37^, 22.3^38^ and 34^39^ degrees, depending on the method used to measure the curvature. No significant sex differences are noted but increase in lordosis with age is evident^37, 40^. Cervical lordotic curvature distributes the compressive load differently from rest of the spine; 36% of compressive load is transmitted through the anterior column and 64% through the posterior facet joints^41, 42^.

Configuration of the sagittal cervical curve has important clinical implications^43–45^. Attainment of the depth of cervical lordosis is reported to be an important factor in achieving better surgical outcomes in patients with neurologic deficits^46–50^, and therefore, rehabilitation of cervical lordosis is considered as an important part of treatment as the compression of nervous tissue can be injurious otherwise^51^.

McAviney *et al*.^52^ have found increase in the number of cervical complaints below 31 and above 40 degree of cervical lordosis. They have also noticed that patients with straight and/or kyphotic cervical curves were 18 times more likely to report symptoms. A statistically significant association has also been found between cervical lordosis of less than 20 degree and cervical pain^52^. Harrison *et al*.^53^ have reported that chronic pain was associated with lowest lordotic curves in 70 patients with chronic neck pain. Studies have also found an association between depth of cervical lordosis and chronic headache^54, 55^. Several researchers have reported that neurologic improvement is associated with posterior shift of the spinal cord and for this to happen, lordotic alignment of the cervical spine is essential to allow the spinal cord to shift dorsally^46, 56, 57^.

In the present study, we have found that improvement in JOA/mJOA score at the latest follow-up was significantly higher in patients who underwent discectomy and corpectomy in comparison with posterior surgery subjects which was associated with significantly higher change in lordosis in the anterior surgery subjects. In a previous meta-analysis comparing anterior decompression and fusion with laminoplasty in the treatment of multilevel cervical ossification of posterior longitudinal ligament, laminoplasty group was found to have significantly reduced postoperative cervical lordosis which was also associated with late deterioration of neurological function^58, 59^.

In the present study, higher changes in surgical outcomes are seen in individuals with more severe disease conditions preoperatively e.g. change in JOA score at latest follow-up was strongly inversely associated with the preoperative JOA/mJOA score (coefficient: −0.74; p = 0.00001). There was also a significant positive correlation between the change in JOA/mJOA score and change in lordosis (r = 0.575; p = 0.002) at the latest follow-up. Similarly, there was significant inverse correlation between the change in lordosis and change in neck VAS score (r = −0.591; p = 0.0158) at the latest follow-up. This may suggest that surgery is better equally for patients with moderate or severe conditions. Whether patients with less or moderate conditions may be advised to seek non-surgical treatments first should depend on the availability and suitability of non-surgical treatment options and risks involved.

Success of cervical laminectomy as well as laminoplasty is determined by the presence of lordosis angle of at least 10 degrees and the preservation of at least 50% facet joints^60, 61^. Laminoplasty involves two distinct effects; a direct effect of posterior decompression and an indirect effect of anterior decompression due to posterior shift of the spinal cord from anterior compressive lesions^62^ and therefore, is considered as a useful alternative. However, laminoplasty is associated with poor clinical outcomes in the presence of kyphotic or S-shaped cervical spine malalignment owing to the prevention of posterior shift of the spinal cord^63^. Although, in the present study, change in lordosis at the latest follow-up had no significant association with the degree of preoperative cervical curvature; neither in anterior nor in posterior surgery groups, data were relatively lesser for studying this effect in posterior surgery group. Moreover, it is reported that cervical lordosis does not significantly differ between asymptomatic and symptomatic individuals and there is considerable inter-individual variation in the lordotic degree of the spinal spine^55, 64^.

In the present study, it was not possible to examine the effect of surgical interventions on the cervical range of motion owing to the unavailability of adequate data. However, in a separate meta-analysis of 36 studies, we have found that not only the cervical ROM decrease significantly after spondylosis surgery but also there was a significant positive relationship between decrease in ROM and follow-up duration (unpublished data). Other studies have also reported decrease in ROM after cervical spondylosis surgery^65, 66^.

In the present study, only a few studies reported adjacent segmental complications a synthesis of which revealed approximately 4.1 ± 2.7% incidence. Among other notable surgery-related adverse events included implant dislodgement (5.7 ± 5.1%), dysphagia (11.2 ± 8.4%), dysphonia (7 ± 4.4%), hematoma (2.2 ± 1.4%), CSF leakage (3.4 ± 5.7%), nerve palsy (5.12 ± 4.6%), and neurological complications (5 ± 3.6%). Previously, it is reported that the majority of complications with cervical spondylosis surgery are well-manageable and have no long-term impact^67^. However, an increased risk of complications has been found to be associated with later age, higher operative time, and combined anterior-posterior procedures^67^.

Among the limitations of the present study, one constraint was that relatively lesser data regarding the quality of life after surgery and at follow-ups were available. None of the studies measured SF36 PCS/MCS scores. There can also be some impact of the lordosis measurement method as it is found that Cobb method slightly underestimates cervical lordosis than posterior tangent method^53^. In the individual studies of the present meta-analysis, all investigators measured lordosis by using Cobb method. Use of the random effects model for the meta-analyses may also affect the precision of effect sizes. However, it was necessary to choose because of the inclusion of more heterogenous populations with majority of the studies were retrospective in design. Because, among the included studies, 2 were randomized controlled, 2 were prospective non-randomized and rest were retrospective in design, the present study was also hampered by the avoidance of the quality assessment with a single tool.

## Conclusion

Cervical spondylosis surgery is found to be associated with significant increase in lordosis as well as JOA/mJOA score in surgeries with anterior approaches but not with posterior surgical approaches. Improvement in lordosis angle was significantly positively associated with follow-up duration and age and inversely associated with the change in lordosis. Independently, gender and preoperative JOA/mJOA score had no significant associations with lordosis change in anterior surgery group, but had significantly inverse relationships with the change in lordosis at the latest follow-up in posterior surgery subjects. In posterior surgery group, after the adjustment of age and gender, only preoperative JOA/mJOA score had significantly inverse relationship with the change in lordosis at the latest follow-up.

## Electronic supplementary material

Supporting Information
