# Supplementary material for: Anterior Cervical Spondylosis Surgical Interventions are Associated with Improved Lordosis and Neurological Outcomes at Latest Follow up: A Meta-analysis
Source: Sci Rep. 2017 Jun 30;7:4407. doi: 10.1038/s41598-017-04311-6 (PMC5493671; doi:10.1038/s41598-017-04311-6)
Supplement: Supplementary file 1 — Supporting Information [file 41598_2017_4311_MOESM1_ESM.doc]

**Anterior Cervical Spondylosis Surgical Interventions are Associated with Improved Lordosis and Neurological**

**Outcomes at Latest Follow up: A Meta-analysis**

Running title: Cervical lordosis after cervical spondylosis surgery

Zengdong Meng^1^, Jing Yu^3^, Chong Luo^1^**,** Xia Liu ^4^, Wei Jiang^2^, Lehua Yu^2^, Rongzhong Huang^2#^

1. Department of Orthopaedics, First People’s Hospital of YunNan Province, YunNan, P. R. China
2. Department of Rehabilitation Medicine, The Second Affiliated Hospital of Chongqing Medical University, Chongqing, P. R.

China

1. Department of Preventive Medicine, Keck School of Medicine, University of Southern California, Los Angeles
2. Shanghai Key Laboratory of Forensic Medicine, Institute of Forensic Science, Ministry of Justice, Shanghai, P.R. China.

**Corresponding address:**

Dr. Rongzhong Huang

Department of Rehabilitation Medicine

The Second Affiliated Hospital of Chongqing Medical University

No. 76 Linjiang Road, Chongqing 400010, China

Tel: +86-18523457722

E-mail: [rzhuang@live.com](mailto:rzhuang@live.com) and rzhuangdoc@hotmail.com

| **Table S1: Characteristics of the included studies** | | | | | | | | | | | | | | | | |
| --- | --- | --- | --- | --- | --- | --- | --- | --- | --- | --- | --- | --- | --- | --- | --- | --- |
| **Study** | **n** | **Followup** | **Design** | **Surgery** | **Technique** | **Age**  **(years)** | **%**  **males** | **Cervical levels involved (%)** | | | | | **Surgery duration**  **(minutes)** | **Blood loss (mL)** | **Hospital**  **Stay**  **(days)** | **%**  **Satisfied** |
|  |  |  |  |  |  |  |  | **1** | **2** | **3** | **4** | **˃4** |  |  |  |  |
| **Burkhadt 2013** | 38 | 20.4±13.7 | RET | CORP | ACCF/ Tit cage | 60.3±11.1 | 66 |  |  |  |  |  |  |  |  | 83 |
| **Burkhadt 2013** | 80 | 20.4±13.7 | RET | DISC | ACDF/ PEEK cage | 60.7±9.9 | 51 |  |  |  |  |  |  |  |  | 86 |
| **Cabraja 2010** | 24 | 35±24.2 | RET | CORP | ACCF | 60.4±9.9 |  | 62 | 0 | 0 | 38 | 38 |  |  |  | 79 |
| **Cabraja 2010** | 24 | 28±13.3 | RET | LMN | Posterior | 66.2±8.8 |  | 0 | 4 | 46 | 50 | 0 |  |  |  | 83 |
| **Chen 2011** | 22 | 48±15.8 | RET | CORP | Tit cage/ autograft fusion | 57.2±7 | 63.6 |  |  |  |  |  |  |  |  | 86.3 |
| **Chen 2011** | 28 | 48±15.8 | RET | LMN | Bone auto grafts fusion | 55.3±5.2 | 67.8 |  |  |  |  |  |  |  |  | 54 |
| **Chen 2011** | 25 | 48±15.8 | RET | LAMP | Open-door LAMP | 54.2±8.7 | 64 |  |  |  |  |  |  |  |  | 24 |
| **Dai 2008** | 29 | 24±15.8 | RCT | DISC | ACDF/ PEEK cage | 45.3±8.5 | 27.6 | 38 | 62 | 0 | 0 | 0 | 77 |  |  |  |
| **Dai 2008** | 33 | 24±15.8 | RCT | DISC | ACDF/ PEEK cage | 45.3±8.5 | 42.4 | 42 | 58 | 0 | 0 | 0 | 54 |  |  |  |
| **Edwards 2002** | 13 | 49±21.2 | RET | CORP | ACCF | 53±7.8 |  |  |  |  |  |  | 224 | 572±165 | 5±2.8 |  |
| **Edwards 2002** | 13 | 40±19.5 | RET | LAMP | LAMP | 54±8.2 |  |  |  |  |  |  | 216 | 360±165 | 3±2.8 |  |
| **Gao 2012** | 145 | 102±15.8 | RET | CORP | ACCF | 51±8.2 | 64.1 | 92 | 8 | 0 | 0 | 0 |  |  |  |  |
| **Kawakami 2002** | 67 | 42±13.4 | RET | LAMP | Expansive | 63.2±12.2 | 80.6 |  |  |  |  |  |  |  |  |  |
| **Kimura 2000** | 8 | 29±9 | RET | LAMP | LAMP | 57±9 | 87 |  |  |  |  |  |  |  |  |  |
| **Kimura 2000** | 31 | 26±10 | RET | LAMP | LAMP | 58±12 | 87 |  |  |  |  |  |  |  |  |  |
| **Lee 2014** | 15 | 24±15.8 | RET | LMN | Alone | 61.3±6.6 | 86.7 |  |  |  |  |  |  |  |  |  |
| **Lee 2014** | 21 | 24±15.8 | RET | LMN | Laminectomy/fusion | 63.7±7.7 | 90.5 |  |  |  |  |  |  |  |  |  |
| **Lee 2014** | 21 | 24±15.8 | RET | LAMP | LAMP | 54.2±10.3 | 71.4 |  |  |  |  |  |  |  |  |  |
| **Lian 2010** | 50 | 31.6±6 | RCT | CORP | Contiguous corpectomy/ fusion/ Titanium cage | 60.8±9.5 | 60 | 0 | 0 | 84 | 16 | 0 | 168±32 | 379±111 |  |  |
| **Liao 2008** | 19 | 12±1.5 | PROS | DISC | ACDF/ PEEK cage | 52.9±10 | 47.4 |  |  |  |  |  | 177±46 |  | 3.8±1.3 | 74 |
| **Lin 2012** | 63 | 24±15.8 | RET | CORP | ACCF | 57.9±10 | 68.3 | 0 | 0 | 81 | 19 | 0 | 125±26 | 149±74 |  | 69.8 |
| **Lin 2012** | 57 | 24±15.8 | RET | DISC | ACDF | 58.7±9.7 | 66.7 | 0 | 0 | 81 | 19 | 0 | 138±31 | 103±51.3 |  | 78.9 |
| **Liu J 2015** | 24 | 86±11 | RET | CORP | ACCF/ Autograft /Allograft | 51.8±5.9 | 37.5 | 100 | 0 | 0 | 0 | 0 | 123±21 | 75±55 |  |  |
| **Liu J 2015** | 22 | 84.5±13 | RET | DISC | ACDF/ Autograft/ allograft | 48.9±9.4 | 45.5 | 0 | 100 | 0 | 0 | 0 | 97±17 | 550±150 |  |  |
| **Liu Y 2012** | 39 | 26.4±8.7 | RET | CORP | ACCF/ Titanium cage | 47.8±6.4 | 66.7 | 0 | 0 | 100 | 0 | 0 | 117±30 | 172±68.2 |  | 54 |
| **Liu Y 2012** | 69 | 26.8±6.8 | RET | DISC | ACDF | 46.1±6.8 | 56.5 | 0 | 0 | 100 | 0 | 0 | 144±32 | 108±49.6 |  | 84 |
| **Machino 2014** | 505 | 25.6±12.6 | RET | LAMP | Double-door LAMP | 66.6±9.4 | 61.2 |  |  |  |  |  | 77±30 | 50±47.5 |  |  |
| **Niu 2010** | 25 | 30.4±3.3 | PROS | DISC | ACDF/ PEEK cage | 52.2±10.5 | 48 | 64 | 36 | 0 | 0 | 0 | 177±49 |  | 4.1±1.1 | 80 |
| **Niu 2010** | 28 | 31.9±3.4 | PROS | DISC | ACDF/ Tit cage | 49.5±11.5 | 53.6 | 68 | 32 | 0 | 0 | 0 | 149±41 |  | 4.3±1 | 75 |
| **Oh 2009** | 17 | 27.3±15 | RET | CORP | ACCF | 55.1±11.6 | 53 |  |  |  |  |  | 210±6 | 779±644 | 17±7.7 |  |
| **Oh 2009** | 14 | 24.9±15 | RET | DISC | ACDF | 52.6±11.6 | 53 |  |  |  |  |  | 141±45 | 306±151 | 15±8.5 |  |
| **Ohashi 2014** | 121 | 24±15.8 | PROS | LAMP | LAMP/foraminotomy | 64±10.2 | 71.9 |  |  |  |  |  | 130±34 | 229±185 |  |  |
| **Ohashi 2014** | 115 | 24±15.8 | PROS | LAMP | LAMP/no foraminotomy | 63.6±12.3 | 70.4 |  |  |  |  |  | 100±33 | 263±296 |  |  |
| **Song 2012** | 15 | 94.3±25.3 | RET | CORP | ACCF/ Autograft/Allograft | 54.1±9.8 | 73.3 | 0 | 67 | 33 | 0 | 0 | 268±65 | 575±265 | 18±7.7 |  |
| **Song 2012** | 25 | 87.3±21.7 | RET | DISC | ACDF/ Autograft/Allograft | 50.3±7.5 | 76 | 0 | 0 | 72 | 28 | 0 | 186±58 | 302±102 | 11±4.1 |  |
| **Abbreviations:** ACCF, Anterior cervical corpectomy and fusion; ACDF, Anterior cervical discectomy and fusion; CORP, corpectomy; DISC, discectomy; LAMP, laminoplasty; LMN, laminectomy; PEEK, Polyetheretherketone; PROS, prospective; RCT, randomized controlled trial; RET, retrospective; Tit, titanium | | | | | | | | | | | | | | | | |

# Table S2: Outcomes of the metaregression analyses with change in lordosis at latest follow-up as dependent variable (all surgery types)

| **Explanatory Variable** | **Coefficient** | **p** | **Datasets** |
| --- | --- | --- | --- |
| Study size | 0.004 [-0.013, 0.021] | 0.629 | 35 |
| Follow-up duration | 0.085 [0.03, 0.14] | **0.004** | 35 |
| Age | -0.338 [-0.57, -0.11] | **0.005** | 35 |
| Gender (% males) | -0.117 [-0.22, -0.02] | **0.022** | 31 |
| Smokers (%) | 0.094 [-0.31, 0.50] | 0.515 | 5 (insuf) |
| % Myelopathy | 0.0004 [-0.047, 0.047] | 0.985 | 31 |
| % Radiculopathy | -0.002 [-0.064, 0.059] | 0.934 | 31 |
| % Radiculomyelopathy | -0.021 [-0.1003, 0.059] | 0.604 | 31 |
| Disease duration (years) | -0.07 [-0.233, 0.098] | 0.339 | 7 (Insuf) |
| 1-level surgery | 0.0004 [-0.07 ,0.07] | 0.99 | 16 |
| 2-level surgery | 0.009 [-0.07, 0.09] | 0.824 | 16 |
| 3-level surgery | 0.008 [-0.054, 0.07] | 0.786 | 16 |
| >3 level surgery | -0.105 [-0.27, 0.06] | 0.195 | 16 |
| Surgery duration | -0.017 [-0.05, 0.02] | 0.316 | 21 |
| Blood loss during surgery | -0.006 [-0.018, 0.005] | 0.26 | 16 |
| Hospital stay | 0.079 [-0.22, 0.38] | 0.552 | 9 |
| Incidence of complication (%) | 0.044 [-0.109, 0.197] | 0.535 | 12 |
| Baseline JOA/mJOA score | -1.30 [-2.35, -0.26] | **0.017** | 26 |
| Baseline NDI score | 0.287 [-0.38, 0.95] | 0.319 | 7 (Insuf) |
| Baseline ROM (degree) | 0.025 [-0.79, 0.84] | 0.936 | 6 (Insuf) |
| Baseline lordosis (degree) | -0.119 [-0.44, 0.20] | 0.455 | 35 |
| Baseline neck VAS score | 1.315 [0.42, 2.21] | **0.008** | 14 |
| Baseline arm VAS score | 2.32 -3.99, 8.63] | 0.254 | 4 (Insuf) |

Abbreviations: mJOA, modified Japanese Orthopedic Association, NDI, neck disability index; ROM, range of neck motion; VAS, visual analogue scale

# Table S3: Outcomes of the metaregression analyses with change in lordosis at latest follow-up as dependent variable (Anterior approaches)

| **Explanatory Variable** | **Coefficient** | **p** | **Datasets** |
| --- | --- | --- | --- |
| Study size | 0.028 [-0.03, 0.09] | 0.326 | 22 |
| Follow-up duration | 0.058[0.001, 0.12] | **0.047** | 22 |
| Age | -0.204 [-0.55, 0.14] | 0.235 | 22 |
| Gender (% males) | 0.0009 [-0.16, 0.16] | 0.99 | 20 |
| Smoking | Insufficient data |  |  |
| % Myelopathy | -0.0002 [-0.049, 0.048] | 0.993 | 20 |
| % Radiculopathy | -0.013 [-0.071, 0.046] | 0.654 | 20 |
| % Radiculomyelopathy | 0.006 [-0.106, 0.118] | 0.908 | 20 |
| Disease duration | Insufficient data |  |  |
| 1-level surgery | -0.015 [-0.07, 0.05] | 0.609 | 15 |
| 2-level surgery | -0.003 [-0.07, 0.07] | 0.93 | 15 |
| 3-level surgery | 0.012 [-0.041, 0.06] | 0.642 | 15 |
| >3 level surgery | 0.017 [-0.18, 0.22] | 0.853 | 15 |
| Surgery duration | -0.027 [-0.067, 0.013] | 0.173 | 17 |
| Blood loss during surgery | -0.009 [-0.021, 0.003] | 0.128 | 12 |
| Hospital stay | Insufficient data |  |  |
| Baseline JOA/mJOA score | -0.505 [-1.88, 0.87] | 0.441 | 15 |
| Baseline NDI score | 0.076 [-0.78, 0.93] | 0.741 | 4 (Insuf) |
| Baseline ROM | Insufficient data |  |  |
| Baseline lordosis angle | 0.067 [-0.29, 0.42] | 0.696 | 22 |
| Baseline neck VAS | 0.43 [-0.79, 1.65] | 0.421 | 8 |
| Baseline arm VAS | 2.32 [-3.99, 8.63] | 0.254 | 4 |
| Incidence of complications (%) | 0.039 [-0.13, 0.21] | 0.62 | 11 |

Abbreviations: mJOA, modified Japanese Orthopedic Association, NDI, neck disability index; ROM, range of neck motion; VAS, visual analogue scale

## Table S4: Outcomes of the metaregression analyses with change in lordosis at latest follow-up as dependent variable (Posterior approaches)

| **Explanatory Variable** | **Coefficient** | **p** | **Datasets** |
| --- | --- | --- | --- |
| Study size | 0.009[-0.005, 0.02] | 0.197 | 13 |
| Follow-up duration | 0.19 [0.01, 0.37] | **0.04** | 13 |
| Age | -0.114 [-0.57, 0.34] | 0.595 | 13 |
| Gender (% males) | -0.182 [-0.37, 0.007] | **0.057** | 11 |
| Smoking | Insufficient data |  |  |
| % Myelopathy | Insufficient data |  |  |
| % Radiculopathy | Insufficient data |  |  |
| % Radiculomyelopathy | Insufficient data |  |  |
| Average number of levels | Insufficient data |  |  |
| 1-level surgery | Insufficient data |  |  |
| 2-level surgery | Insufficient data |  |  |
| 3-level surgery | Insufficient data |  |  |
| >3 level surgery | Insufficient data |  |  |
| Surgery duration | Insufficient data |  |  |
| Blood loss during surgery | Insufficient data |  |  |
| Hospital stay | Insufficient data |  |  |
| Baseline JOA/mJOA score | -1.371 [-2.23, -0.51] | **0.006** | 11 |
| Baseline NDI score | Insufficient data |  |  |
| Baseline ROM | Insufficient data |  |  |
| Baseline lordosis angle | -0.228 [-0.79, 0.33] | 0.389 | 13 |
| Baseline neck VAS | -3.59 [-10.13, 2.95] | 0.202 | 6 (Insuf) |
| Baseline arm VAS | Insufficient data |  |  |
| Incidence of complications (%) | Insufficient data |  |  |

Abbreviations: mJOA, modified Japanese Orthopedic Association, NDI, neck disability index; ROM, range of neck motion; VAS, visual analogue scale
